# EBV Infection Is Common in Gingival Epithelial Cells of the Periodontium and Worsens during Chronic Periodontitis

**DOI:** 10.1371/journal.pone.0080336

**Published:** 2013-12-19

**Authors:** Séverine Vincent-Bugnas, Sébastien Vitale, Caroline C. Mouline, Wafa Khaali, Yves Charbit, Patrick Mahler, Isabelle Prêcheur, Paul Hofman, Janet L. Maryanski, Alain Doglio

**Affiliations:** 1 Université Nice-Sophia Antipolis, UFR Médecine, ImCelVir URE004, Nice, France; 2 Université Nice-Sophia Antipolis, UFR Odontologie, LSBV URE001, Nice, France; 3 Centre Hospitalier Universitaire de Nice, Pôle Odontologie, Hôpital Saint Roch, Nice, France; 4 Centre Hospitalier Universitaire de Nice, Unité de Thérapie Cellulaire et Génique, Nice, France; 5 Centre Hospitalier Universitaire de Nice, Cancéropole PACA, Laboratoire de Pathologie Clinique et Expérimentale, Biobanque IRCAN, Inserm U1081, Nice, France; 6 INSERM, UMR 576, F-06202 Nice, France; University of Toronto, Canada

## Abstract

An amplifying role for oral epithelial cells (ECs) in Epstein-Barr Virus (EBV) infection has been postulated to explain oral viral shedding. However, while lytic or latent EBV infections of oro/nasopharyngeal ECs are commonly detected under pathological conditions, detection of EBV-infected ECs in healthy conditions is very rare. In this study, a simple non-surgical tissue sampling procedure was used to investigate EBV infection in the periodontal epithelium that surrounds and attaches teeth to the gingiva. Surprisingly, we observed that the gingival ECs of the periodontium (pECs) are commonly infected with EBV and may serve as an important oral reservoir of latently EBV-infected cells. We also found that the basal level of epithelial EBV-infection is significantly increased in chronic periodontitis, a common inflammatory disease that undermines the integrity of tooth-supporting tissues. Moreover, the level of EBV infection was found to correlate with disease severity. In inflamed tissues, EBV-infected pECs appear to be prone to apoptosis and to produce larger amounts of CCL20, a pivotal inflammatory chemokine that controls tissue infiltration by immune cells. Our discovery that the periodontal epithelium is a major site of latent EBV infection sheds a new light on EBV persistence in healthy carriers and on the role of this ubiquitous virus in periodontitis. Moreover, the identification of this easily accessible site of latent infection may encourage new approaches to investigate and monitor other EBV-associated disorders.

## Introduction

Epstein-Barr virus (EBV) is a widespread virus responsible for chronic human infections associated with various malignancies, functional abnormalities of immunity, and oral diseases [1]–[3]. EBV persistence requires the establishment of a latent infection within the resting B-cell compartment where viral gene expression is mainly restricted to the non-coding EBV-encoded RNAs (EBERs), viral miRNAs, and various subsets of viral latent genes [1], [3]. The oral cavity is the site for entry and egress of infectious EBV, and viral shedding into saliva is continuous varying from low to high levels [4]. EBV reactivation, with subsequent virus shedding, is thought to occur sporadically in activated tonsilar B-lymphocytes, however one model proposes amplification via infection of epithelial cells (ECs) in the upper aerodigestive tract [4]–[6]. Epithelial EBV infection has been well established using *in vitro* models [7]–[15], and in vivo EBV-infected ECs are commonly encountered in nasopharyngeal carcinoma (NPC) [16], [17], oral hairy leukoplakia (OHL) [18], or acute infectious mononucleosis [5]. In contrast, in healthy individuals, lytic or latent EBV-infected ECs were rarely found in tonsil and tongue sections and none in salivary glands, and evidence that oro/nasopharyngeal ECs actively contribute to EBV replication is scarce [19], [20].

Numerous PCR-based studies have established that EBV-DNA is commonly associated with chronic periodontitis (CP) [21]–[25], a common inflammatory disease recognized as a major cause of tooth loss [26], [27]. During development of CP, resorption of the alveolar bone and deepening of the gingival sulcus crevice leads to the formation of a periodontal pocket (PP) whose depth correlates with disease progression. Intriguingly, the amount of EBV DNA detected in PPs correlates with disease severity [23], [24], [28]–[31]. However, evidence is still lacking to demonstrate that EBV actively replicates in periodontal tissues, and if so, which periodontal cells are infected and which pathogenic mechanisms might be involved.

We hypothesized that the periodontal epitheliums that line the gingival sulcus and attach the teeth to the gingiva, namely the sulcular epithelium (SE) and the junctional epithelium (JE) [32], [33], could represent possible targets for EBV. Using a simple, non-surgical method to sample periodontal tissue, we demonstrate for the first time, not only a widespread EBV-infection of gingival ECs of the periodontium (pECs) during CP, but unexpectedly, a low but detectable level of EBV infection in pECs from healthy sulcus. Moreover, we observe that the level of EBV infection correlates with CP development and promotes cell damage that may increase local inflammatory conditions.

## Results

### Detection of EBV-infected gingival epithelial cells in periodontal epitheliums

**Figure 1:**
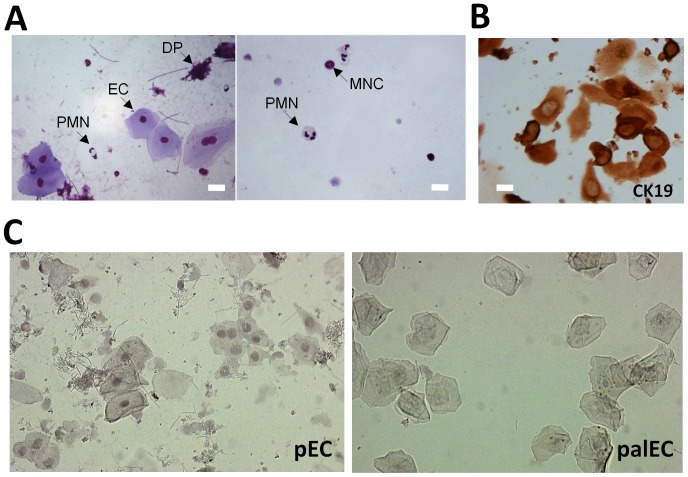
EBER-ISH staining of liquid-based periodontal samples reveals EBV-Infected epithelial cells. (A) Representative MGG staining of cytospin cells collected from PP samples (n = 10). Large spread epithelial-like cells (EC), polymorphonuclear leucocytes (PMN), mononuclear cells (MNC) as well as traces of dental plaque (DP) are indicated with arrows. Size bar represents 15 µM. (B) Representative CK19 staining of pECs from 5 CP patients. Coverslips were processed for IHC with DAB chromogen staining, CK19 specific staining was assessed by comparing with background staining observed using non specific mouse IgGs (not shown). Size bar represents 15 µM. (C) Nuclear EBER-ISH staining in pECs and palECs. EBER-ISH was used to detect EBER in periodontal and palatal cells sampled from 3 patients with chronic periodontitis. Two representative fields (x20) of EBER staining (EBER) are shown for the same selected CP patient with pECs (left panel) and palECs (right panel).

**Figure 2:**
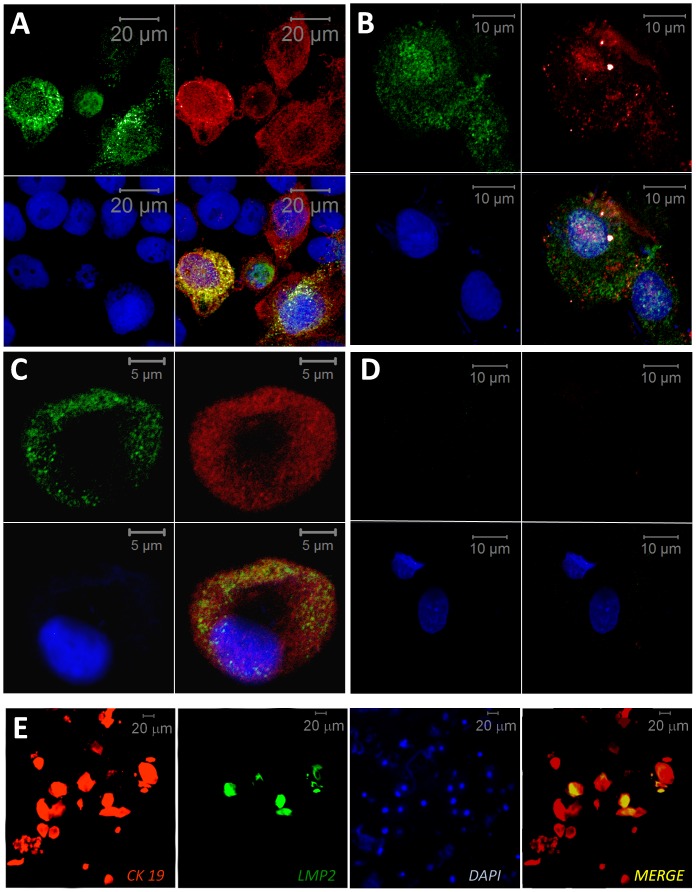
LMP1 and LMP2 IF co-staining in oral epithelial cells. (A–D) Detection of LMP2 and LMP1 by IF double staining in oral epithelial cells, LMP2 (green, upper left), LMP1 (red, upper right), DAPI (blue, below left), merge (below right). (A) Oral TR146 ECs infected with Ad_5_F_35_ recombinant adenoviruses expressing LMP2 and/or ΔLMP1 were used to assess IF-based co-staining of LMP1 and LMP2 in oral epithelial cells. Results show representative specific co-detection of LMP1 and LPM2 in TR146. (B–D) pECs and palECs were sampled from 3 patients with CP and results are shown for the same selected patient. (B, C) Representative LMP1 and LMP2 co-staining in pECs. (D) Lack of LMP1 and LMP2 specific staining in palECs. TR146 were handled and fixed in a manner similar to pECs and palECs. Processing of all cell preparation with two different rat LMP2-specific antibodies (clone 14B7 clone 15F9) showed very similar results, only results obtained with clone 15F9 are presented. Specific staining was assessed by comparing with LMP1 and LMP2 negative ECs, i.e., TR146 cells infected with adenoviral vectors expressing either inactive-LMP1 (Ad_5_F_35_-ΔLMP1) or LMP2 (Ad_5_F_35_-LMP2) (not presented), and EBV-negative palECs. Background staining was also assessed in presence of unspecific rat primary antibodies (not shown). Size-bar is indicated for each panel.

Liquid-based cytology proved feasible for analysis of non-surgical PP samples from patients undergoing routine care for CP. Apart from traces of dental plaque (DP) and occasional polymorphonuclear and mononuclear leucocytes the vast majority of material consisted of large cells (>15 µM) displaying a spread morphology characteristic of ECs (Fig. 1). Cytokeratin (CK) expression in human is site specific, with CK19 and CK4 characteristic of JE and SE, respectively [32], [33], [34], and as expected (Fig. 1 B) most collected pECs (68%±15, n = 5) were from JE (CK19^pos^) and occasional epithelial-like cells were also CK4^pos^ (unpublished data). The presence of EBV-infected cells in periodontal material was first investigated using EBER-ISH in periodontal and palatal samples collected from 3 consecutive CP patients (Fig. 1 C). EBER positive (EBER^pos^) pECs were detected only in PP samples, notably in pECs with large nuclei and high nucleo-cytoplasmic ratio suggesting that EBV infection may preferentially occur in proliferating ECs of the basal epithelial layer. EBV-infection of pECs was then confirmed at the protein level through IF-based confocal microscopy detection of LMP2 and LMP1, two EBV-encoded latent membrane proteins (Fig. 2). Specificity of LMP1 and LMP2 IF co-staining was first assessed using the TR146 cells, a well recognized oral epithelial cell model [35], infected either with Ad_5_F_35_-ΔLMP1 or Ad_5_F_35_-LPM2 (unpublished data), or with Ad_5_F_35_-ΔLMP1/LPM2 (Fig. 2 A). Interestingly, LMP2, and to a lesser extend LMP1, were also specifically detected in clinical samples of pECs (n = 3, Fig. 2 B–C) similarly to pictures from the TR146-based experiments. Higher magnification (Fig. 2 C) showed characteristic LMP2 patches of fluorescence [36] and more diffuse LMP1 cellular distribution. On the contrary, for each CP patient (n = 3), we failed to detect specific LMP2 and/or LMP1 staining in palECs collected from the palatal epithelium in the immediate vicinity of EBV-infected PP (Fig. 2 D). Moreover, LMP2 and CK19 IF co-staining (Fig. 2 E) established that EBV infection occurred notably in CK19^pos^ pECs (n = 5). IF co-staining is difficult to use quantitatively, particularly with heterogeneous clinical samples, nevertheless, we estimated the frequency of CK19^pos^ infected with EBV (LMP2^pos^) in PP from CP patients to range around 32% (±15%, n = 5) of the total of CK19^pos^ pECs.

**Figure 3:**
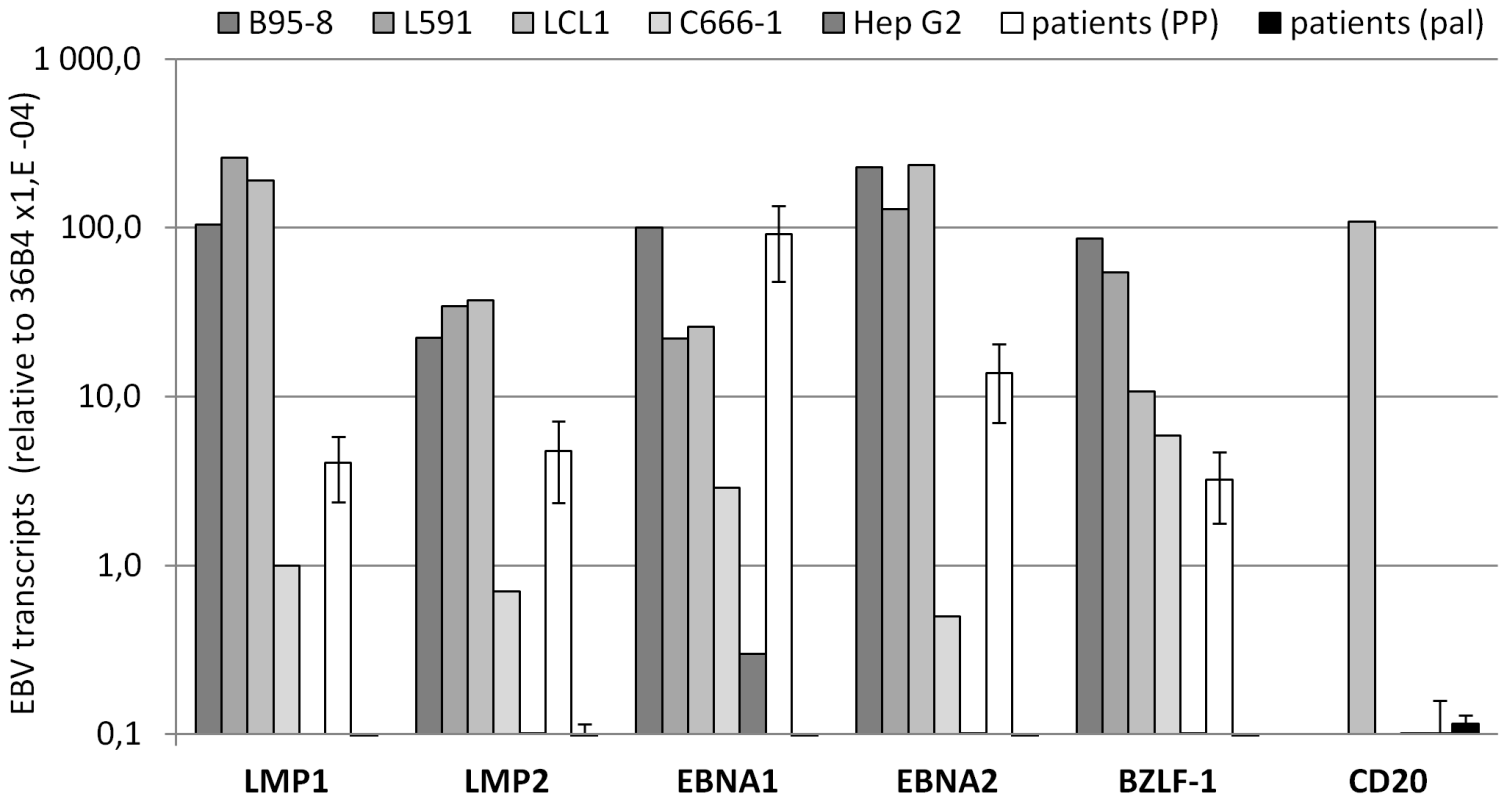
Specific real-time RT-PCR quantification of EBV gene expression in periodontal material. Whole RNA was isolated from 12 periodontal paired-samples (PP) from 6 CP patients, 3 palatal sites from 3 additional CP patients (pal), 4 EBV-infected cell lines (B95-8, L591, LCL1, C666-1) and an EBV-negative cell line (HepG2). The average levels of EBNA1, EBNA2, LMP1, LMP2, and BZLF1 EBV transcripts in clinical samples (shown as mean and SD) were compared with the expression of these EBV-genes in the different cell lines. Values obtained with an EBV-negative cell line were used to determine background level (HDLM2). The average levels of the B-cell marker CD20 transcript were also compared between LCL1, pal, and PP samples. The 36B4 housekeeping gene was used for normalization, with results presented as relative to 36B4 using a LOG_10_ scale. The results shown are from one representative experiment of two RT-PCR experiments performed, each in triplicate.

The presence of latent EBV proteins in pECs indicated that these cells might be latently infected with EBV. To confirm this result, we performed RT-PCR based experiments to detect different EBV transcripts using 12 paired-RNA samples from 6 CP patients (patients 1 to 6, Table 1). EBV-specific latent (LMP1, LMP2, EBNA1, EBNA2) and lytic (BZLF1) transcripts were detected in all PP samples from patients (Fig. 3). The average transcript levels measured in PPs were largely within the range expressed by a series of EBV-infected cell lines (B95-8, L591, LCL1, C666-1) and at least 300 fold higher than background levels established using EBV-negative cell lines (HepG2, HDLM2). Latent EBV transcripts (EBNA1, LMP1, LMP2, EBNA2) were specifically detected in all CP samples, EBNA1 was expressed at higher, or very similar levels to those measured in EBV-infected cell lines, while expression of the other latent EBV genes was lower. Specific EBNA1 RNA expression in pECs was also confirmed using two additional sets of primers (unpublished data) which allowed specific detection of large spliced EBNA1 transcripts initiated either from the W/C or the F EBV promoters [37]. Moreover, the immediate early viral transactivator BZLF-1, known to mediate the disruption of latent EBV infection in inducing the EBV lytic cycle, was expressed in pECs albeit at a level notably lower than that observed in the EBV productive cell line B95-8 (Fig. 3). The lack of EBV infection in palECs was also confirmed at the transcript level since we failed to detect any EBV transcripts in pal RNAs. Finally we excluded the possibility that EBV transcripts detected in PP material could be originated from infiltrating inflammatory EBV-infected B-lymphocytes (CD20^pos^ cells) because the CD20 transcript, used as B cell marker, was never detected above background levels in 10 EBV-infected paired PP RNA samples (patient 1 to 5, Table 1 & Fig. 3). As a positive control, CD20 transcript were easily detected in RNA from the lymphoblastoïd cell lines LCL1 (Fig. 3) and from blood mononuclear cells (unpublished data). In addition, B-lymphocytes were never, or very rarely, detected by IF-based detection of CD20 in periodontal samples material (unpublished data).

**Table 1:** Patients and sample information.^a^

Patients N°	Age (Y)	Sex	Pocket depth^b^ (mm)	Clinical attachment level^b^ (mm)	Gingival index^b^	EBV-infected pECs^c^ (%)	RNA sampling^d^	Tunel analysis^e^
1	75	F	4.2	4.5	2	37	X	
			9.1	9.3	3	41	X	
2	45	M	4.1	4.1	2	28	X	
			5.7	6	2	26	X	
3	70	F	2.5	3.5	1	15	X	
			4.8	6.5	1	34	X	
4	64	M	3.6	4	1	29	X	
			5.5	7.2	2	36	X	
5	58	F	3	3.5	2	16	X	
			7.3	7.5	2	27	X	
6	58	M	3.5	3.8	1	17	X	
			6.4	6.9	2	24	X	X
7	51	F	3.1	3.1	0	18		
			5.3	5.5	2	31		X
8	69	F	4.1	4.6	2	28		
			5.7	5.7	2	5		X
9	64	F	2.5	2.5	1	0		
			6.8	7	2	33		X
10	59	F	3.2	3.2	0	24		
			6.2	6.2	1	34		X
11	36	F	4.2	4.2	0	12		
			6.3	6.3	2	24		
12	44	F	3	3	0	16		
			5.8	6.1	2	27		
13	56	M	2.7	2.9	0	6		
			4.6	5.2	1	9		
14	41	M	3.1	3.1	1	2		
			4.8	4.8	1	0		
15	64	F	3.6	3.6	2	10		
			5	5.7	2	3		
16	56	M	5.1	5.6	2	22		
			7.8	8	2	36		
17	38	F	2.7	2.7	0	28		
			5.2	5.2	2	31		
18	54	M	4	4.5	2	30		
			9	9.2	2	54		
19	56	M	4.3	4.5	1	20		
			6	6	1	35		
20	47	M	3.2	3.4	0	5		
			7.4	7.5	2	19		
**Mean** f	**55.2**		**4.9**	**5.15**	**1.4**	**22.3**		
*SD*	*10.8*		*1.7*	*1.8*	*0.8*	*12.6*		

a Characteristics for the main CP patient cohort used in this study (n = 20).

b For each patient, paired-sampling was performed, and upper and lower values shown correspond to shallow and deep periodontal sites (SS and DS), respectively. Normal clinical attachment level and gingival index values are considered to be less than 3 mm and 1, respectively.

c Frequency (%) of EBV-infected periodontal epithelial cells (pECs). The mean frequency (and standard deviation) of EBV-infected cells was 18.5% (+/−5.12) for SS, and 26.45% (+/−6.8) for DS.

d RNA samples used for EBV and CCL20 gene expression analysis.

e Samples used for TUNEL experiments.

f Overall mean values and standard deviations (SD).

### The extent of EBV infection correlates with periodontitis severity

**Figure 4:**
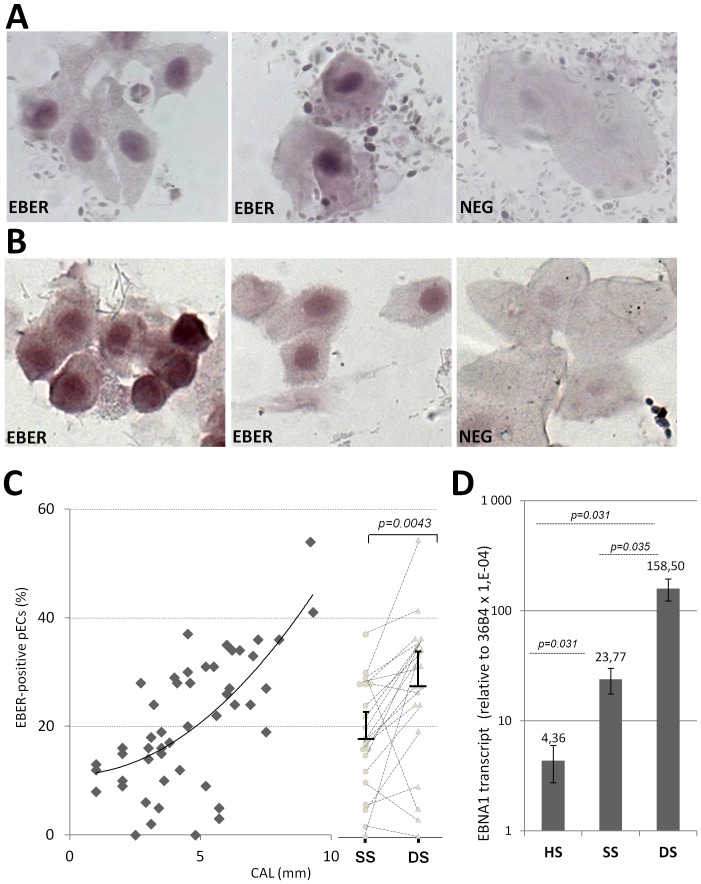
EBV infection increases with disease severity. (A–B) EBER-ISH staining was performed on paired periodontal pocket DS and SS samples (n = 40) from CP patients (n = 20), and from HS samples from healthy gingival sulcus from HDs (n = 10). Two representative fields (x40) of EBER staining (EBER) is shown for (A) one selected CP patient and (B) for one selected HD. Negative controls (NEG) were processed using a random PNA probe, and for each individual, the same cell sampling was used for positive and negative staining. (C) EBER-ISH-based determination of the frequency of EBV-infected pECs (EBER^pos^) in periodontal samples from healthy sites (HS), swallow sites (SS), and deep sites (DS). The graph (left part) shows the tendency curve of EBER^pos^ pECs and the clinical attachment level (CAL) in 40 paired-samples from 20 CP patients and 10 samples from healthy donors (same as in A and B). The dot-plot analysis (right part) shows the comparative analysis of the frequency of EBER^pos^ pECs in paired-samples (SS circles, DS triangles) collected from 20 CP patients (same samples as on graft). (D) The average levels of EBNA1 transcript were also compared between whole RNA from HS (n = 10) and 12 paired-samples (SS and DS, n = 6 for each) from 6 CP patients (same samples as in C). The 36B4 housekeeping gene was used for normalization, with results presented as relative to 36B4 using a LOG_10_ scale. The p values were calculated using Wilcoxon signed-rank test.

The presence of EBV infected-cells in periodontal material was then investigated using EBER-ISH in a series of 40 paired-samples sampled at 2 paired periodontal sites (SS and DS) from 20 CP patients (Table 1). Nearly all samples (38/40) contained ECs that showed specific EBER nuclear staining (Fig. 4 A) with a global mean of 22.3% (±12.6%, n = 40) (Table 1). Interestingly, EBER-ISH analysis performed on samplings from 10 healthy donors (HDs) (Fig. 4 B) demonstrated that all sampled healthy sites (HS) from HDs also contained EBV-infected pECs with a significant and reproducible frequency (13.2%±3.49, Table 2). Overall, analyzing the EBER^pos^ pEC frequency measured in CP patients and HDs (Tables 1 and 2), we found a clear positive correlation with the level of disease progression as measured by deterioration in clinical attachment levels (CAL) (Fig. 4 C left). Moreover, a paired-statistical analysis established on paired-samplings of PP cells collected from SS and DS showed that the frequency of EBER^pos^ pECs was significantly higher in DS (26.45%±6.8, n = 20) than in SS (18.5%±5.12, n = 20) (Fig. 4 C right and Table 1, p = 0.0043). The relationship between CP severity and EBV infection was also confirmed at the transcript level using paired-statistical analysis of EBNA1 expression in DS and SS from 6 CP patients (Fig. 4 D, p = 0.031). EBNA1 transcripts were also specifically detected in HS, however the mean level of EBNA1 expression was about 5-fold and 36-fold lower in HS compared to SS and DS, respectively, from CP patients with very significant p values (Fig. 4 D).

**Table 2:** Healthy donors and sample information.^a^

Donors n°	Age (Y)	Sex	Pocket depth^b^ (mm)	Clinical attachment level^b^ (mm)	Gingival index^b^	EBV-infected pECs^c^ (%)	RNA sampling^d^
1	32	M	1	1	1	13	x
2	30	F	1	1	0	8	x
3	45	M	3	3,5	1	19	x
4	31	F	2	2	0	16	x
5	29	F	2	2	0	16	x
6	27	M	3	3	0	14	x
7	26	M	2	2	0	9	x
8	28	M	2	2	1	10	x
9	33	M	1	1	0	12	x
10	44	M	2	2	0	15	x
**Mean** e	**32,5**		**1,9**	**1,95**		**13,2**	
*(SD)*	*6.7*		*0.7*	*0.8*		*3,49*	

a Characteristics for the healthy donors (HDs) used in this study (n = 10).

b For each patient, one sampling was performed in healthy gingival sulcus of the right mandibular molar. Normal Clinical attachment level and gingival index values are considered to be less than 3 mm and 1, respectively.

c Frequency (%) of EBV-infected periodontal epithelial cells (pECs).

d RNA samples used for EBNA1 and CCL20 gene expression analysis.

e Overall mean values and standard deviations (SD).

### Epithelial cell death and production of the inflammatory chemokine CCL20 are associated with EBV infection

**Figure 5:**
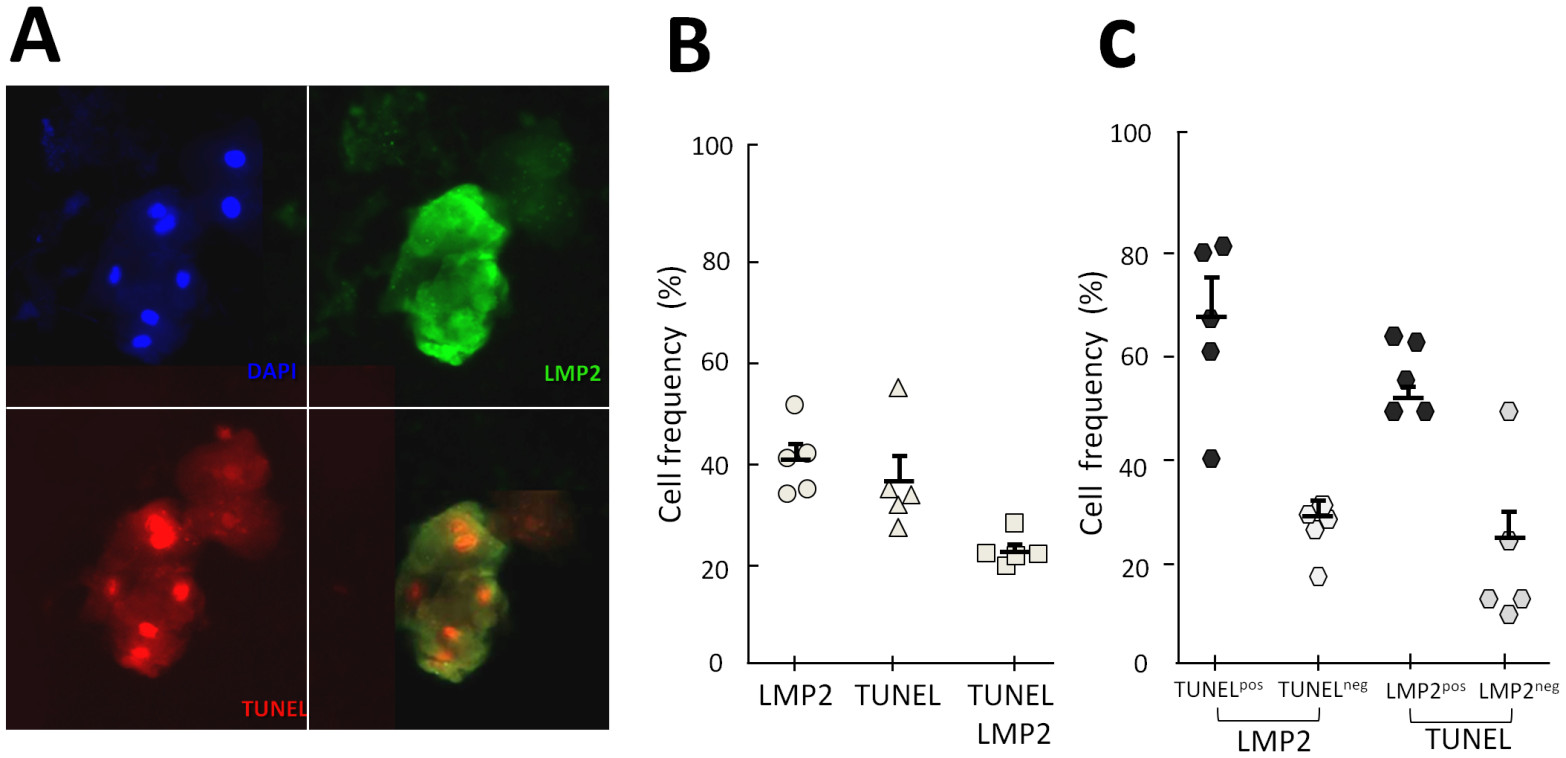
Infection of periodontal epithelial cells by EBV is associated with apoptotic cell death. TUNEL assay associated with IF LMP2 detection was used to identify apoptotic cells and EBV-infected cells in DS samples from 5 CP patients. (A) Representative double fluorescent staining with antibodies specific for LMP2 (upper right panel), TUNEL staining (below left panel), with the latter two merged (below right panel). The upper-left panel shows DAPI staining of nuclei in the same field (x20). (B) Quantitative evaluations of A. Cell counting of LMP2^pos^ (LMP2), TUNEL^pos^ (TUNEL) and double positive (LMP2 & TUNEL) pECs (n = 5). Data represent the frequency of positive pECs identified in each category. (C) Calculations from B showing the frequency of apoptotic pECs (TUNEL^pos^) among EBV-infected pECs (LMP2) (left part) and the frequency of EBV-infected pECs (LMP2^pos^) among apoptotic pECs (TUNEL) (right part). Mean and standard deviation is shown for each group.

Previous data have described the frequent presence of apoptotic epithelial cells in PP [38]. Because in our histological analysis we also commonly identified apparently apoptotic ECs with diminished cytoplasm, we used a fluorescent TUNEL assay in conjunction with IF staining for LMP2 to investigate the relationship between EBV infection and epithelial apoptosis (Fig. 5). Using 5 representative samples collected from DS (patients 6 to 10, Table 1), we found that around 38% of pECs were apoptotic cells (TUNEL^pos^), and that a significant part (23.2%±1.5) of TUNEL^pos^ pECs were also infected with EBV (Fig. 5 B). Pearson's Chi-square analysis showed that apoptosis and EBV-infection of pECs were strongly associated (n = 307 p<10^−9^), indicating that EBV-infected pECs were more likely to be apoptotic than non-infected pECs, and that, apoptotic pECs were more likely to be EBV-infected compared to non-apoptotic pECs (Fig. 5 C).

**Figure 6:**
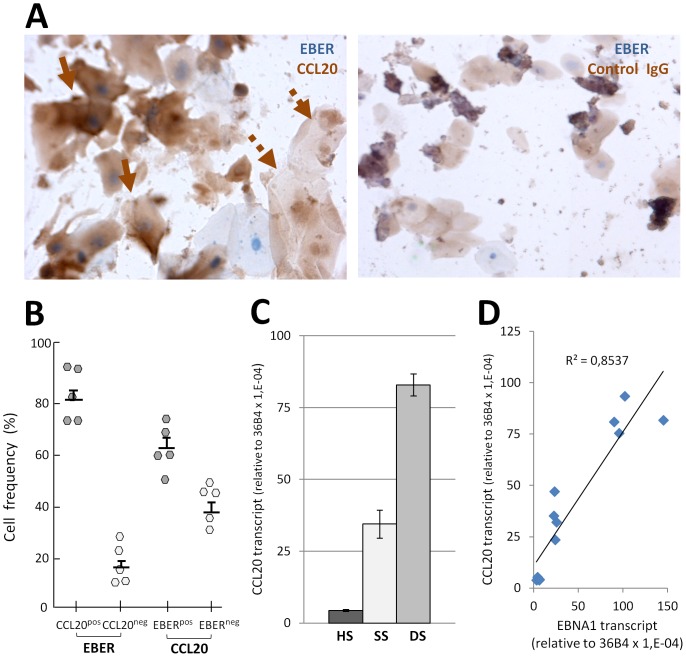
Production of the inflammatory chemokine CCL20 by EBV-infected periodontal epithelial cells. (A) Left panel shows representative CCL20 staining of pECs from 5 CP patients (DS samples) (HIS, x40) of EBV-infected cells (EBER-ISH) (solid arrows) and of EBV-negative pECs (dotted arrows). Right panel shows background staining observed using nonspecific goat IgGs. (B) Quantitation of (A) (n = 5). The frequency (%) of CCL20^pos^ pECs among EBV-infected pECs (EBER, left part) and of EBER^pos^ pECs among CCL20-producing pECs (CCL20, right part) is shown. Mean and standard deviations are shown for each group. (C) Real-time RT-PCR quantification of CCL20 transcripts in whole RNA isolated from 12-paired RNA samples from 6 CP patients (SS n = 6, DS n = 6) and 2 RNA samples from healthy donors. (D) Real-time RT-PCR quantification of EBNA1 and CCL20 transcripts in whole RNA isolated from CP patients (n = 8) and from healthy donors (n = 2). Graph shows the linear tendency curve of CCL20 related to EBNA1. Simple linear regression analysis showed a positive correlation. Data are representative of 2 independent experiments, each performed in triplicate. The 36B4 housekeeping gene was used for normalization. The results are presented as relative to 36B4 (×10^−6^).

Moreover, we then investigated whether EBV may worsen local inflammatory conditions by promoting production of CCL20, a pivotal inflammatory chemokine known to be up-regulated in EBV-infected tumors [17], [39], [40]. Histological analysis (Fig. 6 A and B), combining EBER-ISH and CCL20 staining, established that CCL20 could be detected in the vast majority (∼80%) of EBER^pos^ pECs in 5 CP samples (patients 1 to 5, Table 1) but was very rare in 2 HS samples (not shown). However in CP patients, CCL20 production was apparently not restricted to EBV-infected cells since 38% of EBER-negative pECs also stained positive for CCL20 (Fig. 6 B). Nevertheless, by the Pearson's Chi-squared test established that EBV-infection and CCL20 production by pECs were strongly associated in CP patients (p<10^−38^). Moreover, detection of CCL20 RNA in RNA samples from HS, SS, and DS (n = 2, 6 and 6 respectively) confirmed that CCL20 was highly produced in DS but not in HS (Fig. 6 C). Interestingly, the level of CCL20 RNA expression correlated closely with that of EBNA1 (Fig. 6 D, R^2^ = 0.8537).

## Discussion

In the present work, using a simple cell-sampling procedure to investigate EBV infection of periodontal tissues we made the surprising discovery that ECs from periodontal tissues are commonly infected with EBV at a frequency that varied from an average of 13.2% in healthy sulcus to more than 30% in the deepest PPs of patients with CP. Our findings are therefore novel in that they establish *in vivo* a model of epithelial EBV infection in otherwise healthy individuals. In addition, they support the involvement of EBV in periodontitis by establishing for the first time that periodontal epitheliums may serve as privileged targets for EBV, and that the extent of EBV infection in these epitheliums closely correlates with severity of CP.

First, we found that EBV-Infected pECs are likely to be in a state of latent EBV infection. This conclusion is mainly supported by the profile of EBV gene expression in periodontal material showing consistent expression of latent transcripts (i.e. EBNA1, EBNA2, LMP2 and LMP1) and of typical latent EBV proteins (i.e. LMP1, LMP2). Moreover, the lytic transactivator BZLF-1 is detected to very low level, which, overall, is even below the low level observed in latently infected NPC cells C666-1. It has been known for a long time that there is a pool of latently EBV-infected B-cells in the healthy carrier, but the consistent presence of a pool of latently-infected oral ECs is a completely new observation and alters current paradigms. The pool of latently EBV-infected pECs is probably of large size if one takes into account the entire dentition since the total area of periodontal epithelium is thought to be over several square cm. Our discovery of an oral reservoir of EBV-infected epithelial cells in healthy individuals may have implications for the spread of EBV since this commonly found site might serve as a significant source for salivary viral shedding. At this stage, since low but detectable level of BZLF-1 transcripts are present in our samples, one cannot exclude the occurrence of transient lytic cycle bursts in this large population of EBV-infected ECs and possibly viral amplification/activation of EBV to spread within the epithelium [4], [12]. This point might be important to understand consistent virus shedding in the saliva of healthy carriers and will require further investigation.

Second, the high frequency of EBV-infected pECs establishes that periodontal epitheliums, namely the JE and the SE, are permissive to EBV infection. Interestingly, JE is a unique stratified non-keratinized epithelium distinguishable from other epitheliums by its pattern of integrins and cytokeratins expression, high rate of cell turnover, and notably wide intercellular spaces harboring neutrophils and mononuclear leucocytes [34], [38]. The highly permeable tissue architecture of the JE may facilitate EBV infection of pECs via direct transfer from infiltrating EBV-coated B cells, as shown thus far only *in vitro* [12]. Interestingly, a large number of B cells, likely infected with EBV, were observed in the connective tissue subjacent to the gingival epithelium of patients with periodontal disease [25]. Moreover, EBV utilizes different glycoprotein complexes to enter B cells and ECs. The initial attachment to B cells is mediated by the interaction of EBV glycoprotein gp350/220 with CD21, whereas the integrins integrins α5β6, α5β8, and α5β5 appear to function as EBV receptors on epithelial cells[13]. Interestingly, in contrast to other gingival epitheliums, JE express α5β6 [41] making ECs from JE likely permissive for EBV infection. Whether the presence of a specific type of integrin may explain EBV viral tropism in oral epitheliums remains to be investigated.

Last, for a long time a relationship has been suspected between EBV and CP because PCR-based studies have established that levels of EBV-DNA detected in PP were correlated with disease severity [23], [25], [28]–[31]. Our results establish that EBV is already present in pECs before the onset of periodontitis and that the extent of EBV-infection in pECs is increased with periodontitis severity. The presence of latently EBV-infected pECs in healthy sulcus may represent a baseline of infection, and subsequent changes in the gingival microenvironment may alter the balance to increase EBV infection. In turn, EBV may exacerbate inflammatory disease by inducing cell death and promoting a proinflammatory response in infected cells. Previous reports have established that JE and SE contain more apoptotic cells than other gingival epitheliums [38], [42]. High frequency of apoptotic pECs may compromise the JE integrity, promoting gingival detachment and deepening the periodontal pocket; it may also pave the way for bacterial colonization by increasing adherence to epithelial cells and/or by allowing release of “danger signals” by injured cells to favor biofilm development [26], [43], [44]. Whether EBV is directly responsible of periodontal EC death, through direct or indirect mechanisms such as T-cell mediated killing of infected cells, and to what extent EBV contributes to epithelial breakdown remain important questions to be elucidated in future studies. Concurrently, leucocyte infiltration and inflammatory mediators induced by the host response appear to play a critical role in periodontal inflammation [27], [43]. The chemokine (C-C motif) receptor 6 (CCR6)/CCL20 receptor/ligand axis provides key homing signals recruiting CCR6^pos^ CD4+ Th17 or regulatory T-cells that drive pro-inflammatory or suppressive immune responses, respectively [45]. Intriguingly, Hosokawa et al. [46] showed that both CCL20 and CCR6^pos^ CD4 T-cells are found at high frequency in inflamed periodontal tissue. EBV may thus contribute to modify the immune environment by triggering regulatory T-cell infiltration in inflamed periodontal lesions through CCL20 production. Interestingly, CCL20 was easily detectable in CP samples but not in those from HDs, indicating that CCL20 is only upregulated in inflamed conditions. Intense production of CCL20 has been previously reported in EBV-infected cells from NPC and HL with a direct stimulating role for EBNA1 established at least in HL [17], [39], [40]. Therefore, it is tempting to speculate that the EBNA1 up-regulation we show in CP patients may directly activate CCL20 production during CP.

Identifying EBV as a *bona fide* periodontic pathogen is of importance to lay a foundation for better management of this common chronic disease. It will be important to find ways to distinguish between EBV contributing to the pathogenesis of the periodontitis, and EBV infection of pECs being a consequence of the inflammatory conditions. In the context of a synergistic model between periodontic bacteria and viruses, such information may greatly help toward a better understanding of the respective contribution of each pathogen during periodontitis development. In this context, an encouraging but only single-case study reported that EBV load decreased and severity of the periodontal condition improved after antiviral treatment [47]. Our study should thus serve as an impetus toward further clinical trials and the search for new antiviral drugs. Since periodontitis can aggravate other, often lifestyle-related inflammatory conditions, more effective management could have a broad impact on public health.

## Materials and Methods

### Donors and periodontal charting

Patients were diagnosed for CP at the periodontal department of the University Hospital of Nice accordingly to classification of the periodontal diseases [48]. They had no history of periodontal treatment and no antibiotic therapy for at least 6 months before inclusion. The exclusion criteria included heart disease, diabetes mellitus, human immunodeficiency virus infection, inflammatory bowel disease, immunosuppression, liver or kidney dysfunction, oral mucosal inflammatory condition (lichen planus, leukoplakia, oral cancer). Disease severity was evaluated with the periodontal pocket depth (distance between the gingival margin and the bottom of the pocket), the clinical attachment level (CAL: distance between the cemento-enamel junction and the bottom of the pocket), and the gingival index. Probing was carried out using a Williams probe calibrated in millimeters and pocket depths and CALs were assessed by the same examiner at four sites per tooth, mesiofacial, midfacial, distofacial and midlingual, for all teeth in the oral cavity (complete periodontal charting) [49]. Sampling of CP patients and healthy donors was done during scheduled visits for conventional non-surgical periodontal therapy benefiting from local anesthesia (articaïne, 1/200 000) administered for root planing. A series of 10 consecutive patients was established to set up pEC and palaltal EC (palEC) sampling. Relationship with disease progression was analyzed with PP samples from a cohort of 20 CP patients (9 men, 11 women, aged 36–75), and for each patient paired PP samples were prepared for measurements of the frequency of EBV-infected pECs, RNA analysis, LMP2 and TUNEL- (terminal deoxynucleotidyltransferase (TdT)-mediated dUTP nick end labeling) analysis and CCL20-based investigations (Table 1). It was not logistically possible to obtain EBV serologic screening of CP patients since EBV serology is not required for routine dental care. Additionally, 10 healthy volunteer donors (HDs, 7 men, 3 women, aged 26–45) undergoing routine scaling were selected as free of any periodontal diseases after periodontal charting (Table 2). All subjects recruited in this study read and understood the information note and signed the informed consent. Samples were collected with approval of the Nice University Hospital institutional review board (sample collection N° DC-2009-1012).

### Sampling

For all PP sampling, the site was isolated from saliva with cotton rolls and air-dried, supragingival plaque was removed with a scaler, and then a sterile periodontal curette was gently inserted to the bottom of the periodontal pocket in order to remove by a single stroke the subgingival material. For 6 consecutive CP patients, in addition to PP sampling, palatal ECs (palEC) were also collected from the keratinized epithelial palatal surface immediately adjacent to the anaesthetized upper molar where the PP sampling was done. Before collecting palECs, the upper keratinized anucleated epithelial layer was gently removed with a sterile curette. Three of these patients were used for EBER staining and whole RNA isolation and 3 patients for confocal microscopy experiments. For the CP patient cohort (n = 20), paired PP samples were prepared based on pocket-depth measurements performed at 4 sites per tooth for the whole dentition (see above). One sample was from the shallowest pocket (shallow site; SS) and the other from the deepest pocket (deep site; DS) found. In practice, these corresponded to the pockets from two different teeth, rather than two from the same tooth. For the HD cohort, a single sample was prepared for each donor from a healthy sulcular site (HS) located in the right mandibular molar. Healthy gingival sulcus was gently rubbed with a sterile periodontal curette then processed similarly to CP patients. CCL20-based investigations were performed with only two HDs (donors 1 and 2, Table 2). Cell samples were suspended in 500 µL of RPMI 1% FBS and centrifuged at 1,500 rpm for 5 min. The pellet was resuspended in variable volume of PBS 1% FBS (200 to 600 µL) depending on the number of subsequent histological analyses. For histological studies, 250 µL was loaded into a cytospin cuvette (EZ Single cytofunnel, Shandon) and centrifuged at 800 rpm for 10 min onto glass coverslips. Samples were then fixed in 3% paraformaldehyde for 10 min at room temperature and kept at 4°C in PBS for a maximum of one week. For RNA analysis, biological specimens were collected with a sterile periodontal curette and placed in RNA later RNA stabilization reagent (Qiagen) and kept at 4°C. To obtain both histological and RNA samples from the same site, two periodontal curettes were used successively.

### Histologic, immunochemical, molecular, and in situ hybridization studies

MGG staining was performed as recommended (Polysciences). Immunofluorescent staining (IF) were performed as follows, paraformaldehyde fixed-sections were permeabilized in TBS, 0.5% Triton X-100, 1% BSA for 15 min at room temperature, after a saturation step of 1 h in TBS containing 10% donkey serum, sections were incubated overnight at 4°C in a humid chamber with specific primary antibodies diluted at optimized concentration in antibody diluent buffer with background reducing components from Dako furnisher. Primary antibodies were from mouse for CK4 (Abcam), CK19 (Neomarkers), CD20 (Dako), EBV LMP1 (DAKO clones CS1-4) and from rat for two different antibodies against EBV LMP2 (AbD Serotec clone 14B7, Abcam clone 15F9). After 3 wash steps (TBS, 0.25% Tween 80), Alexa-Fluor 594 donkey anti-mouse or donkey anti-rat Alexa-Fluor 488 (Invitrogen) diluted in antibody diluent buffer with background reducing components (Dako) were incubated as secondary antibodies for 45 min at room temperature. After 3 wash steps, nuclei were then identified by DAPI staining, and mounted in Mowiol 4–88 (Polysciences). LMP1 and LMP2 IF co-staining were validated using human TR146 cells, an epithelial cell model commonly recognized as a representative for the human gingival mucosa [35]. Subconfluent TR146 cells were infected (or not) with replication-incompetent adenoviruses vectors expressing either inactive-LMP1 (Ad_5_F_35_-ΔLMP1), or LMP2 (Ad_5_F_35_-LPM2), or both inactive-LMP1 and LMP2 separated by an internal ribosomal entry site (Ad_5_F_35_-ΔLMP1/LPM2) [50] at a vp∶cell ratio of 50,000∶1. All recombinant adenoviruses vectors were produced by the Gene Vector Laboratory of CAGT (Baylor College, Houston, Texas). After 24 h, cells were collected by scraping, and processed similarly to pECs through centrifugation onto glass coverslips using a cytospin cuvette (EZ Single cytofunnel, Shandon) and fixation in 3% paraformaldehyde for 10 min. For LMP2 IF staining very similar results were observed using two different LMP2 antibody clones (15F9 or 14B7), 15F9 was usually preferred for lower background.

EBV-infected cells were also identified using the PNA EBER-in situ hybridization (ISH) detection kit from Dako and EBERs probe set (Dako). Formaldehyde-fixed samples were treated with proteinase K for 30 min, and incubated with EBER PNA probe at 55°C for 80 min and then processed as recommended by the supplier. For double-staining experiments, EBER-ISH-treated coverslips were air-dried and the cells were permeabilized in 100 µl of TBS (50 mM Tris.HCl, pH 7.4 and 150 mM NaCl) containing 0.2% Triton X-100. Permeabilized coverslips were then processed for immunohistochemistry (IHC) using the EnVision Detection System (Dako) following supplier's instructions. Primary antibodies diluted in TBS 1% BSA were incubated overnight at 4°C on coverslips; CCL20 goat antibody (R&D Systems), followed by incubation with the Polymer/HRP reagent (Dako). The reaction was visualized by DAB chromogen (Dako).

For EBER ISH, the background level was checked using, a negative probe composed of random PNA probe provided by the supplier (Dako). In addition the absence of staining in similar neighboring cells, or in EBV-negative palatal ECs, or in control TR146 cells, was used to certify the lack of unspecific background. For immunochemical staining, the background level was checked using irrelevant primary mouse, rat or goat IgGs (Santa Cruz). Furthermore, the absence of staining in similar neighboring cells, or in EBV-negative palatal ECs, or in control TR146 cells was used to certify the lack of unspecific background. Specificity of IF-based staining of LMP1 and LMP2 was assessed using TR146 infected with Ad_5_F_35_-ΔLMP1/LMP2 as positive control, or with Ad_5_F_35_-ΔLPM1 or Ad_5_F_35_-LPM2 as negative control for LMP2- and LMP1-staining, respectively.

Positive staining (EBER and IF staining) was only considered in cells showing an epithelial cell morphology as large spread cells ranging from 15 to 65 µM with central shaped nuclei and high cytoplasm/nuclei ratio. Only individual epithelial cells were counted and very small cells showing dark nuclei staining and atypical cell morphology or cells appearing as overlapping groups of cells were excluded. Acquisition and processing of IF-based confocal analysis were performed using ZEISS LSM 5 Exciter confocal microscope using Zen 2009 acquisition software and ICH and other IF-staining were performed using Axio Observer.Z1 microscope using AxioVision 4.8 acquisition software and digital AxioCam ICc1 Rev.4 camera (Zeiss) and photometrics Cascade II 1024 camera (Photometrics).

### RNA isolation and RT-PCR analysis

After periodontal or palatal sampling and cell centrifugation the weighed-pellet was solubilized in 1% β-mercaptoethanol lysis buffer (350 µl for 3.4 mg of material) and processed for RNA isolation according to manufacturer's instructions (RNeasy Plus Micro kit®, Qiagen). RNAs were also isolated from primary blood mononuclear cells (PBMCs) and from 5 EBV-infected cell lines; C666-1 (carcinoma nasopharyngeal), B95-8 (Marmoset EBV-productive cells), LCL1 (EBV-immortalized B-cells), L591 (EBV^pos^ Hodgkin's lymphoma (HL), and two EBV-negative cell lines; HDLM2 (EBV^neg^ Hodgkin's lymphoma), HepG2 (epithelial cell line). RNA quantification was performed using a microvolume spectrophotometer (Thermo Scientific NanoDrop 2000). Extracted RNA was treated with RNase-free DNase then subjected to amplification by using QuantiFast SYBR® Green RT-PCR (Qiagen). PCR experiments were performed using an ABI PRISM 7000 System (Applied Biosystems). Reactions were performed in a 25 µL final volume using 50 ng of diluted RNA. Amplification conditions were as follows: 50°C, 10 min; 95°C, 5 min; (95°C, 10 sec; 60°C, 30 sec) cycled 40 times. Each sample was run in triplicate. EBV transcripts were detected by RT-PCR using specific sets of EBV primers as previously described [51]. Detection of large spliced EBNA1 transcripts expressed from C/W and F promotors was performed as described [37] with primer sets Y3/K2and Q1/K2, respectively. RT-PCR detection of CD20 transcripts was done using quantiTech primer assay from Qiagen (MS4A1 human gene) and detection of CCL20 transcripts was done as previously reported [46]. The housekeeping gene Acidic Ribosomal Phosphoprotein 36B4 was used as an internal standard in all RT-PCRs experiments (Forward: 5′-TGCATCAGTACCCATTCTATCAT-3′, Reverse: 5′-AGGCAGATGGATCAGCCAAGA-3′). Relative levels of mRNA expression were calculated according to the delta-delta Ct method by normalization to the expression of the 36B4 gene. Positive and negative RNA controls were included in all runs. DNA-free water served as a negative control in each PCR run.

### Detection of apoptotic cells

Apoptotic cells were identified with terminal deoxynucleotidyl transferase (TdT)-mediated dUTP nick end labeling (TUNEL) assay using Click-iT® TUNEL Alexa Fluor 594 Imaging Assay as recommended by the manufacturer (Invitrogen). Briefly, slides of PP cells were incubated 20 min at room temperature in permeabilization reagent (0.25% triton X-100 in PBS), and processed as recommended for visualization of transferase activity with Alexa Fluor 594 chromogen. For double staining experiments, slides were first processed for TUNEL analysis then processed for IF staining of LMP2. Positive and negative controls of nuclear DNA fragmentation furnished by the manufacturer (Invitrogen) were included for assay calibration and specificity assessment.

### Cell counting and statistical analysis

Cell counting was performed by two independent investigators by light or fluorescent microscopy (X40 or X20). The cell number was calculated as the average of 5 randomly selected fields, and fields containing less than 5 cells were not considered. Epithelial cell morphology was easily identified as either very large spread cells ranging from 30 to 65 µM, showing rather angular cell morphology with low nucleo-cytoplasmic ratio characteristic of the well-differentiated ECs from upper epithelial layer, or as smaller spread cells (15 to 30 µM) with central shaped nuclei and higher nucleo-cytoplasmic ratio characteristic of ECs of the basal epithelial layer. Only individual ECs were counted and small cells showing dark nuclei staining and atypical cell morphology or cells appearing as overlapping groups of cells were excluded. Tendency curve between frequency of pECs and CAL was established using least-squares polynomial regression analysis with the best coefficient of determination (r^2^ = 0.45). Comparative statistical analysis between paired groups of cells was performed using Wilcoxon signed-rank test for paired samples. A chi-square test served for statistical analysis of CCL20 and TUNEL linkage to EBV presence. Statistical analysis were performed using BiostaTGV website (http://marne.u707.jussieu.fr/biostatgv/). Results from 5 individual patients (Table 1) were pooled; for TUNEL we included a total of 307 pECs (n = 35, 44, 50, 63, 115 respectively), and for CCL20 we included a total of 610 pECs (n = 107, 116, 124, 124, 139 respectively).
